# Cardiovascular safety of tiotropium Respimat vs HandiHaler in the routine clinical practice: A population-based cohort study

**DOI:** 10.1371/journal.pone.0176276

**Published:** 2017-04-21

**Authors:** Francesco Trotta, Stefania Spila-Alegiani, Roberto Da Cas, Maja Rajevic, Valentino Conti, Mauro Venegoni, Mariangela Rossi, Giuseppe Traversa

**Affiliations:** 1 Department of Epidemiology, Lazio Regional Health Service, Rome, Italy; 2 Pharmacoepidemiology Unit, National Centre for Epidemiology, National Institute of Health, Rome, Italy; 3 PhD Course, Pharmaceutical Sciences, Drug Chemistry and Technologies Department, “La Sapienza” University of Rome, Rome, Italy; 4 Regional Center for Pharmacovigilance, General Directorate for Health, The Lombardy Region, Milan, Italy; 5 Unit for Pharmaceutical Governance, General Directorate for Health, Umbria Region, Perugia, Italy; Kaohsiung Medical University Hospital, TAIWAN

## Abstract

The cardiovascular safety of tiotropium Respimat formulation in the routine clinical practice is still an open issue. Our aim was to compare the risk of acute myocardial infarction and heart rhythm disorders in incident users of either tiotropium Respimat or HandiHaler. The study population comprises patients aged ≥45 years, resident in two Italian regions with a first prescription of tiotropium (HandiHaler or Respimat) between 01/07/2011-30/11/2013. The cohort was identified through the database of prescriptions reimbursed by the Italian National Health Service. Comorbidities and clinical outcomes were obtained from hospital records. The primary outcome was the first hospitalization for acute myocardial infarction and/or for heart rhythm disorders during the exposure period. Hazard ratios were estimated in the propensity score-matched groups through Cox regression. After matching, 31,334 patients with incident prescription of tiotropium were included. The two groups were balanced with regard to baseline characteristics. Similar incidence rates of the primary outcome between Respimat and HandiHaler users were identified (adjusted hazard ratio 1.02, 95% CI 0.82–1.28). No risk difference between Respimat and HandiHaler emerged when considering clinical events separately. This large cohort study showed a comparable acute cardiovascular safety profile of the two tiotropium formulations.

## Introduction

Tiotropium bromide, a long-acting anticholinergic agent widely used in Italy in the management of chronic obstructive pulmonary disease (COPD) is available in two formulations with different inhalation device (HandiHaler or Respimat) [1–4].

A safety signal of cardiovascular adverse events associated with the use of tiotropium Respimat was raised [5,6], even though the data were conflicting. A meta-analysis of clinical trials and an observational cohort study showed a statistically significant increase in the risk of all-cause mortality and cardiovascular death associated with Respimat when compared to HandiHaler [7,8]. However, a large company-sponsored RCT (TIOSPIR) compared head to head the two formulations demonstrating no risk difference on a “hard” outcome such as mortality [9].

Although part of the scientific community acknowledged the trial results, safety concerns were still pending due to the selected patient population enrolled in the trial which may negatively affect its external validity [5,10–14].

More specifically, TIOSPIR trial excluded patients with moderate to severe renal impairment (tiotropium has a primary renal excretion after pulmonary absorption) and patients with cardiovascular diseases, especially in case of unstable or recent conditions (since it was biologically plausible a pro-arrhythmic effect of tiotropium) [15]. Also patients with severe respiratory disorders, including those with a recent COPD exacerbation were excluded. Glaucoma was also a condition who led to trial exclusion (because of the systemic cardiovascular effect of antiglaucoma drugs) [16]. The majority of TIOSPIR patients were already on treatment with tiotropium and this population may have a higher tolerance to anticholinergic effects.

Furthermore, a re-examination of TIOSPIR data highlighted a significant risk of acute myocardial infarction (AMI) by grouping two Respimat arms (fatal AMI, RR: 3.48 95% CI 1.04–11.68; fatal and non-fatal AMI, RR: 1.37, 1.00–1.88) [17].

Accumulating evidence shows that up to 80% of COPD patients in clinical practice are treated according to findings of trials from which they would have been excluded [18]; patients enrolled in company-sponsored COPD trials were younger, predominantly male, with worse lung function and quality of life than COPD patients in routine clinical practice [19]. In addition, tiotropium users in clinical practice are more severe than those included in randomized controlled trials [20]. A real-life drug-utilization study conducted in Germany found that only 30% of COPD patients treated with tiotropium could have been included in the TIOSPIR trial, thus questioning the true benefit/risk profile of the two tiotropium formulation [21].

For these reasons we conducted an observational cohort study aimed at comparing the cardiovascular safety of the two tiotropium formulations, i.e. Respimat and HandiHaler, in a large patient population from the real life setting.

The primary objective of this study was to compare the risk of AMI or heart rhythm disorders in incident users of either Respimat or HandiHaler. The secondary objective was to focus on a composite outcome which included all cardiovascular events, cerebrovascular events, or heart rhythm disorders not already included in the primary outcome.

## Materials and methods

### Study design

We conducted a retrospective cohort study among incident users of tiotropium Respimat and tiotropium HandiHaler in the period 2011–2013.

### Study population

The study population included all patients aged x 45 years, resident in the Umbria Region (around 900,000 inhabitants) and in the Lombardy Region (around 10 million inhabitants) who received at least one prescription of tiotropium (ATC code R03BB04, Respimat or HandiHaler) within the National Health Service between 1 July 2011 and 30 November 2013. Only subjects who received the first prescription of tiotropium (incident users used as a proxy of tiotropium-naïve patients) during the study period were included. We excluded all patients who had received a prescription of tiotropium in the previous 6 months of 2011 and all users who had received both formulations at the first prescription. The date of the first prescription was considered as the index date.

### Data source

All pharmaceutical prescription data derive from the regional monitoring database of medicines reimbursed by the NHS. The database includes prescriptions issued by general practitioners and dispensed to outpatients from local community pharmacies or, in some cases, directly from local health units. Data on over the counter drugs and on drugs dispensed to patients during a hospitalization were not available.

From each prescription, the following information is available at regional level: marketing authorization code (indicating the specific tiotropium formulation, i.e. Respimat or HandiHaler); number of packages; patient code (which is anonymized before any subsequent use); date of the dispensing.

For each formulation we retrieved the information on the "defined daily doses" (DDD). Through record linkage between the anonymized patient code and the regional database of NHS enrollees we retrieved demographic information (age and sex). Information on comorbidities and clinical outcomes were obtained from hospital discharge records (SDO), in which are recorded all the episodes of hospitalization in ordinary and day-hospital regime.

Information included in data sources are property of each Region. The authors received a restricted data set to conduct the study upon specific request and after the submission of the study protocol.

### Exposure

For each formulation, the duration of use was defined according to the expected duration of the prescription (in DDDs), as the total time that the patient is taking the medication, i.e. from the date of first prescription, and of all consecutive prescriptions dispensed, plus two weeks of grace period.

### Definition of the events and follow-up

Study events were all the admissions to hospital for cardiovascular and cerebrovascular events, and heart rhythm disorders coded according to the international classification of diseases 9th revision (ICD-9). Cardiovascular events included the following ICD-9 codes:410-Acute myocardial infarction; 411-Other acute ischemic heart disease; 413-Angina pectoris; 414-Other chronic ischemic heart disease; 425-Cardiomyopathy; 428-Heart failure; 429-Ill-defined heart disease; 798-Sudden death, cause unknown; procedures code 36-Operations on vessels of heart; Cerebrovascular events included all ICD-9 diagnoses codes from 430 to 438), and heart rhythm disorders were identified using ICD-9 diagnoses code 426-Conduction disorders; 427-Cardiac dysrhythmias; procedures code 37-Heart rhythm procedures) (for further details see S1 Table). Diagnostic codes were retrieved from the main diagnosis) from the regional archive of hospital discharges. Only in case of procedures ("operations on vessels of heart" and "heart rhythm procedures") both main and secondary diagnosis codes were retrieved. No information was available on patients admitted to hospital outside the Umbria or the Lombardy Regions.

All subjects with at least one prescription of tiotropium were followed from the first prescription up to the earliest of the following dates: end of the duration of use (exposure period); change in the formulation (switch); hospitalization for study events in the exposure period; in-hospital death for any cause; end of the study (31 December 2013).

The primary outcome was defined as AMI (ICD-9 diagnoses code 410) or heart rhythm disorders (ICD-9 diagnoses code 426; 427; procedures code 37); the secondary outcome included all study events with exclusion of those considered as the primary outcome (i.e. non-AMI cardiovascular events and cerebrovascular diseases).

### Potential confounders

We considered as potential confounders demographic characteristics (age and sex), selected comorbidities and known or suspected risk factors for cardiovascular diseases or severity of respiratory diseases. At least two prescriptions of the same drug category or a hospitalization (main or secondary ICD-9 codes) during the 12 months preceding the index date were considered as a proxy of comorbidities and risk factors. The concomitant use of respiratory medications (ATC codes R03 or H02) before starting tiotropium was considered as a proxy of the severity of the respiratory disease. We also defined a patient population with more severe respiratory diseases as subjects presenting both at least two previous respiratory drugs and a previous hospitalization for exacerbation of COPD and/or pneumonia. The S2 Table gives details on the specific confounders considered in the study.

### Statistical analysis

A descriptive analysis was conducted to compare the characteristics of patients treated with tiotropium Respimat and tiotropium HandiHaler by using a t test for continuous variables and a χ^2^ test for categorical ones.

Given the large number of potential confounders we estimated the propensity score, using a logistic regression model where the dependent variable was the exposure (tiotropium) and the independent variables were all the confounding variables (S2 Table). To control for confounders, Respimat users were matched to HandiHaler user (1:1) based on propensity score. We estimated the propensity score separately in each Region to take into account differences in prescription attitudes. The nearest neighbor matching algorithm was used (caliper width 0.01 of standard deviation of the logit score). To evaluate the performance of the matching procedure, the standardized differences between the groups were estimated (a variable was considered balanced if the standardized difference was below 0.10).

To estimate the impact of tiotropium Respimat and tiotropium HandiHaler on the outcome, we applied a Cox proportional hazard regression comparing hazard rates (HR) of primary and secondary outcomes in the two matched groups of tiotropium users (propensity score-matched cohort). For each patient the observation was censored at the end of the follow-up period (end of the duration of use or change in the formulation or end of the study).

We also conducted several subgroup analyses to evaluate hazard ratios in selected patient populations with clinical characteristics that represented exclusion criteria in the TIOSPIR trial (S2 Table), and that might be considered as potential underlying risk factors for cardiovascular events.

Several pre-planned sensitivity analyses were performed: i) Kaplan-Meier cumulative incidence for primary outcomes by tiotropium formulations; ii) risk of primary outcome in Respimat vs HandiHaler by calendar period (2011–2013); iii) risk of primary outcome in Respimat vs HandiHaler by fixed follow-up period (30, 45 or 75 days following the first prescription of tiotropium, i.e. the index date); iv) risk of in-hospital mortality by tiotropium formulations.

We took the decision to conduct the study in the overall population of the Lombardy and Umbria regions, without selecting of any specific group of patients; no formal estimate of the sample size was performed before data collection.

All statistic tests were two sided. STATA 11 software was used to perform the analysis.

### Ethics committee approval

The study was approved by the ethics committee of the Italian National Institute of Health (Prot. PRE-C-17/15).

## Results

**Fig 1:**
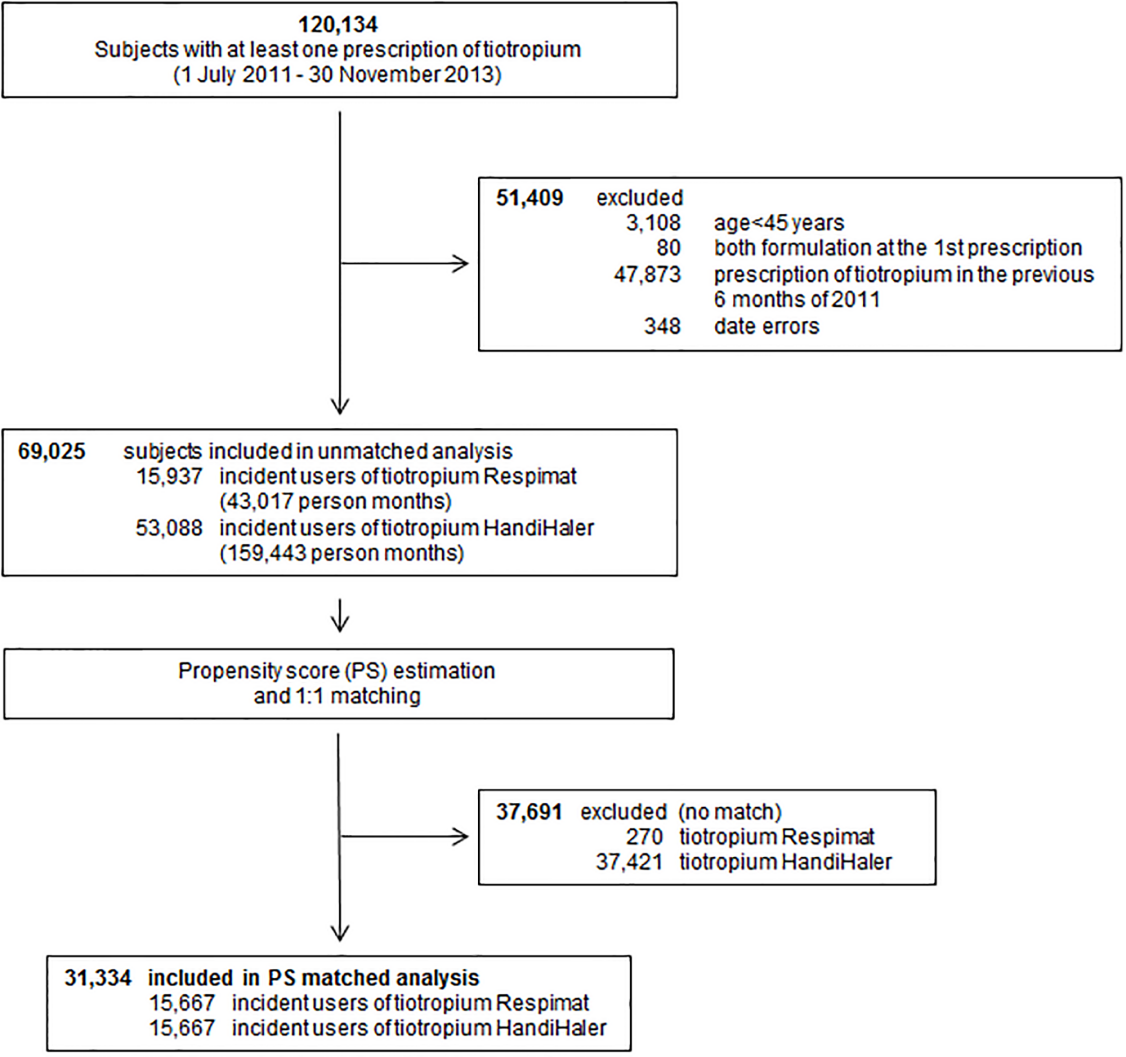
Flow chart of the tiotropium users (Respimat or HandiHaler) enrolled in the study cohort.

During the study period, a total of 120,434 subjects received at least a prescription of tiotropium in the Lombardy and Umbria Regions. We excluded 51,409 subjects mainly because they were not incident users (n. 47,873, 93.1%). The remaining study cohort of 69,025 subjects included 15,937 users of Respimat (23%), corresponding to 43,017 person-months and a median exposure of 45 days, and 53,088 users of HandiHaler (77%), corresponding to 159,443 person-months and a median exposure of 75 days (Fig 1).

Compared with HandiHaler users, Respimat users differed in many characteristics: they were slightly younger, with lower prevalence of both identified comorbidities and drug utilization, and presented a less severe respiratory disease at baseline (Table 1).

**Table 1:** Characteristics and risk factors before and after matching. Values are numbers (percentages) unless stated otherwise.

Characteristics		Before matching		After matching		Standardised difference
		Respimat (No 15,937)	HandiHaler (No 53,088)	Respimat (No 15,667)	HandiHaler (No 15,667)	
**Mean (SD) age, years**		72.2 (10.8)	73.5 (10.6)	72.2 (10.8)	72.3 (10.7)	0.009
**Gender**	Females	7,191 (45.1)	23,400 (44.1)	7,049 (45.0)	7,024 (44.8)	0.003
	Males	8,746 (54.9)	29,688 (55.9)	8,618 (55.0)	8,643 (55.2)	0.000
**Region**	Umbria	1,396 (8.8)	4,830 (9.1)	1,259 (8.0)	1,259 (8.0)	-
	Lombardy	14,541 (91.2)	48,258 (90.9)	14,408 (92.0)	14,408 (92.0)	-
**Hospitalization (at least one episode in the previous 12 months)**	Acute myocardial infarction	351 (2.2)	1,693 (3.2)	345 (2.2)	367 (2.3)	0.009
	Acute ischemic heart disease	114 (0.7)	505 (1.0)	114 (0.7)	112 (0.7)	0.002
	Angina pectoris	833 (5.2)	3,639 (6.9)	819 (5.2)	775 (4.9)	0.013
	Cardiomyopathy	162 (1.0)	811 (1.5)	161 (1.0)	161 (1.0)	0.000
	Arrhythmia	1,028 (6.5)	4,515 (8.5)	1,007 (6.4)	1,065 (6.8)	0.015
	Heart rhythm procedures	288 (1.8)	1,264 (2.4)	287 (1.8)	316 (2.0)	0.013
	Heart failure	1,138 (7.1)	5,527 (10.4)	1,114 (7.1)	1,147 (7.3)	0.008
	Ill-defined heart disease	37 (0.2)	170 (0.3)	37 (0.2)	42 (0.3)	0.006
	Cardiogenic shock	12 (0.1)	48 (0.1)	11 (0.1)	10 (0.1)	0.002
	Diseases of pulmonary circulation	231 (1.4)	958 (1.8)	228 (1.5)	221 (1.4)	0.004
	Diabetes mellitus	2,903 (18.2)	10,949 (20.6)	2,867 (18.3)	2,693 (17.2)	0.029
	Acute kidney failure	136 (0.9)	579 (1.1)	133 (0.8)	145 (0.9)	0.008
	Chronic kidney disease	416 (2.6)	1,898 (3.6)	408 (2.6)	405 (2.6)	0.001
	Neoplasms	885 (5.6)	3,279 (6.2)	872 (5.6)	856 (5.5)	0.004
	Respiratory diseases, excl. COPD	898 (5.6)	3,913 (7.4)	881 (5.6)	816 (5.2)	0.018
	COPD	1,333 (8.4)	6,336 (11.9)	1,310 (8.4)	1,292 (8.2)	0.004
	Disorders of lipoid metabolism	100 (0.6)	412 (0.8)	100 (0.6)	91 (0.6)	0.007
	Operations on vessels of heart	304 (1.9)	1,426 (2.7)	300 (1.9)	277 (1.8)	0.011
	Venous thromboembolism	95 (0.6)	336 (0.6)	91 (0.6)	87 (0.6)	0.003
	Hypertensive disease	1,337 (8.4)	5,620 (10.6)	1,310 (8.4)	1,278 (8.2)	0.007
	Cerebrovascular diseases	621 (3.9)	2,737 (5.2)	614 (3.9)	572 (3.7)	0.014
	Transient mental disorders	1,154 (7.2)	4,286 (8.1)	1,123 (7.2)	1,135 (7.2)	0.003
	Dementias and/or anti-dementia drugs	276 (1.7)	1,026 (1.9)	271 (1.7)	248 (1.6)	0.012
	Parkinson's disease	377 (2.4)	1,311 (2.5)	372 (2.4)	347 (2.2)	0.011
	Epilepsy	44 (0.3)	173 (0.3)	44 (0.3)	48 (0.3)	0.005
	Glaucoma	909 (5.7)	2,909 (5.5)	886 (5.7)	770 (4.9)	0.033
	Pneumonia	765 (4.8)	3,470 (6.5)	749 (4.8)	713 (4.6)	0.011
**Drug use (at least two prescriptions in the previous 12 months)**	Antiarrhythmics class I and III	1085 (6.8)	3,833 (7.2)	1,071 (6.8)	1,006 (6.4)	0.017
	Beta blockers	4,088 (25.7)	13,600 (25.6)	4,017 (25.6)	3,937 (25.1)	0.012
	Antihypertensives and/or diuretic	4,390 (27.5)	17,012 (32)	4,310 (27.5)	4,140 (26.4)	0.024
	Dihydropyridine CCB	2,921 (18.3)	9,985 (18.8)	2,872 (18.3)	2,824 (18.0)	0.008
	Non dihydropyridine CCB	755 (4.7)	3,021 (5.7)	742 (4.7)	640 (4.1)	0.032
	Angiotensin receptor blockers and ACE-I	8,105 (50.9)	28,402 (53.5)	7,989 (51.0)	7,973 (50.9)	0.002
	Other cardiac drugs	1,970 (12.4)	7,850 (14.8)	1,941 (12.4)	1,904 (12.2)	0.007
	Lipid lowering drugs	4,427 (27.8)	15,459 (29.1)	4,366 (27.9)	4,318 (27.6)	0.007
	Anticoagulants	7,110 (44.6)	25,329 (47.7)	7,022 (44.8)	6,768 (43.2)	0.033
	Antidepressants	2,064 (13.0)	7,095 (13.4)	2,022 (12.9)	2,002 (12.8)	0.004
	Opioids	1,484 (9.3)	5,130 (9.7)	1,454 (9.3)	1,397 (8.9)	0.013
	NSAIDs	2,497 (15.7)	8,628 (16.3)	2,455 (15.7)	2,320 (14.8)	0.024
	Glucocorticoids	2,106 (13.2)	6,614 (12.5)	1,963 (12.5)	1,735 (11.1)	0.045
	Anticholinergics	705 (4.4)	5,167 (9.7)	677 (4.3)	641 (4.1)	0.011
	LABA (Salmeterol; Formoterol; Indacaterol)	859 (5.4)	2,632 (5.0)	808 (5.2)	698 (4.5)	0.033
	SABA (Salbutamol; Terbutaline; Fenoterol)	1,179 (7.4)	3,813 (7.2)	1,102 (7.0)	959 (6.1)	0.037
	Leukotriene receptor antagonists	499 (3.1)	1,281 (2.4)	415 (2.6)	376 (2.4)	0.016
	Xanthines	468 (2.9)	1,871 (3.5)	455 (2.9)	385 (2.5)	0.028
	Adrenergics in combination with corticosteroids or other drugs, excl. anticholinergics	4,286 (26.9)	13,869 (26.1)	4,141 (26.4)	3,926 (25.1)	0.031
	Adrenergics in combination with anticholinergics	62 (0.4)	159 (0.3)	49 (0.3)	31 (0.2)	0.023
	Other systemic drugs for obstructive airway diseases	17 (0.1)	30 (0.1)	12 (0.1)	9 (0.1)	0.007
	Corticosteroids for systemic use	2,345 (14.7)	7,448 (14)	2,214 (14.1)	1,993 (12.7)	0.041
	Antibacterials^	11,349 (71.2)	36,712 (69.2)	11,098 (70.8)	11,070 (70.7)	0.004
**Number of prescription respiratory drugs in previous 12 months**§	0	8,684 (54.5)	28,458 (53.6)	8,657 (55.3)	9,041 (57.7)	0.049
	1	1,953 (12.3)	7,271 (13.7)	1,920 (12.3)	1,903 (12.1)	0.003
	2	2,708 (17.0)	8,339 (15.7)	2,682 (17.1)	2,542 (16.2)	0.024
	3	1,493 (9.4)	5,330 (10.0)	1,432 (9.1)	1,406 (9.0)	0.006
	≥4	1,099 (6.9)	3,690 (7.0)	976 (6.2)	775 (4.9)	0.056
**Severity of respiratory disease**~		766 (4.8)	3,518 (6.6)	746 (4.8)	668 (4.3)	0.011

No: number; SD: standard deviation; IQ range: interquartile range; DDD: defined daily dose; COPD: chronic obstructive pulmonary disease; CCB: calcium channel blocker; ACE-I: angiotensin converting enzyme inhibitor; NSAIDs: non-steroidal anti-inflammatory drugs; LABA: long-acting beta agonist; SABA: short-acting beta-agonist

^ at least 1 prescription in the previous 12 months

^§^ number of prescriptions of glucocorticoids and/or anticholinergics and/or laba and/or saba and/or leukotriene receptor antagonists and/or xanthines and/or adrenergics in combination with corticosteroids or other drugs and/or adrenergics in combination with anticholinergics and/or antiallergic agents and/or other systemic drugs for obstructive airway diseases and/or corticosteroids for systemic use

^~^ at least two previous respiratory drugs and a previous hospitalization for exacerbation of COPD and/or pneumonia

After 1:1 matching by propensity score, an overall cohort of 31,334 incident users of tiotropium was available for the analysis and the two groups can be considered balanced with respect to baseline characteristics.

During the period of tiotropium use, among a total of 2,722 incident events we identified 797 primary events: 152 in Respimat users (incidence rate = 0.95 x 100 person-months) and 645 in HandiHaler users (incidence rate = 1.21 x 100 person-months). The analysis in the propensity score-matched cohort showed similar incidence rates of primary outcome between Respimat and HandiHaler users (adjusted HR 1.02, 95% CI 0.82–1.28) (Table 2 and S1 Fig). A similar risk was also observed for the secondary outcome (adjusted HR 0.88, 95% CI 0.76–1.02). No risk difference between Respimat and HandiHaler emerged when grouping all the events considered in the study (adjusted HR 0.92, 95% CI 0.81–1.04), for AMI (adjusted HR 1.04, 95% CI 0.70–1.55) and for heart rhythm disorders (adjusted HR 1.00, 95% CI 0.78–1.31) analyzed separately (Table 2).

**Table 2:** Hazard ratio of primary and secondary outcomes for patients treated with Respimat versus HandiHaler.

Outcomes	Unmatched cohort			Propensity score-matched cohort		
	No (%) of cases		Unadjusted Hazard ratio (95% CI)	No (%) of cases		Adjusted Hazard ratio (95% CI)
	Respimat No 15,937	HandiHaler No 53,088		Respimat No 15,667	HandiHaler No 15,667	
Primary outcome	152 (0.95)	645 (1.21)	0.86 (0.72–1.02)	151 (0.96)	164 (1.05)	1.02 (0.82–1.28)
*AMI*	*48 (0*.*35)*	*184 (0*.*35)*	*0*.*94 (0*.*69–1*.*30)*	*47 (0*.*30)*	*50 (0*.*32)*	*1*.*04 (0*.*70–1*.*55)*
*Hearth rhythm disorders*	*107 (0*.*67)*	*477 (0*.*90)*	*0*.*82 (0*.*67–1*.*01)*	*107 (0*.*68)*	*119 (0*.*76)*	*1*.*00 (0*.*78–1*.*31)*
Secondary outcome	325 (2.04)	1,600 (3.01)	0.73 (0.65–0.82)	321 (2.05)	400 (2.55)	0.88 (0.76–1.02)
All events	477 (2.99)	2,245 (4.23)	0.77 (0.69–0.85)	472 (3.01)	564 (3.60)	0.92 (0.81–1.04)

No: number; %: proportion of patients with events; CI: confidence interval; AMI: acute myocardial infarction

### Subgroup analyses

**Fig 2:**
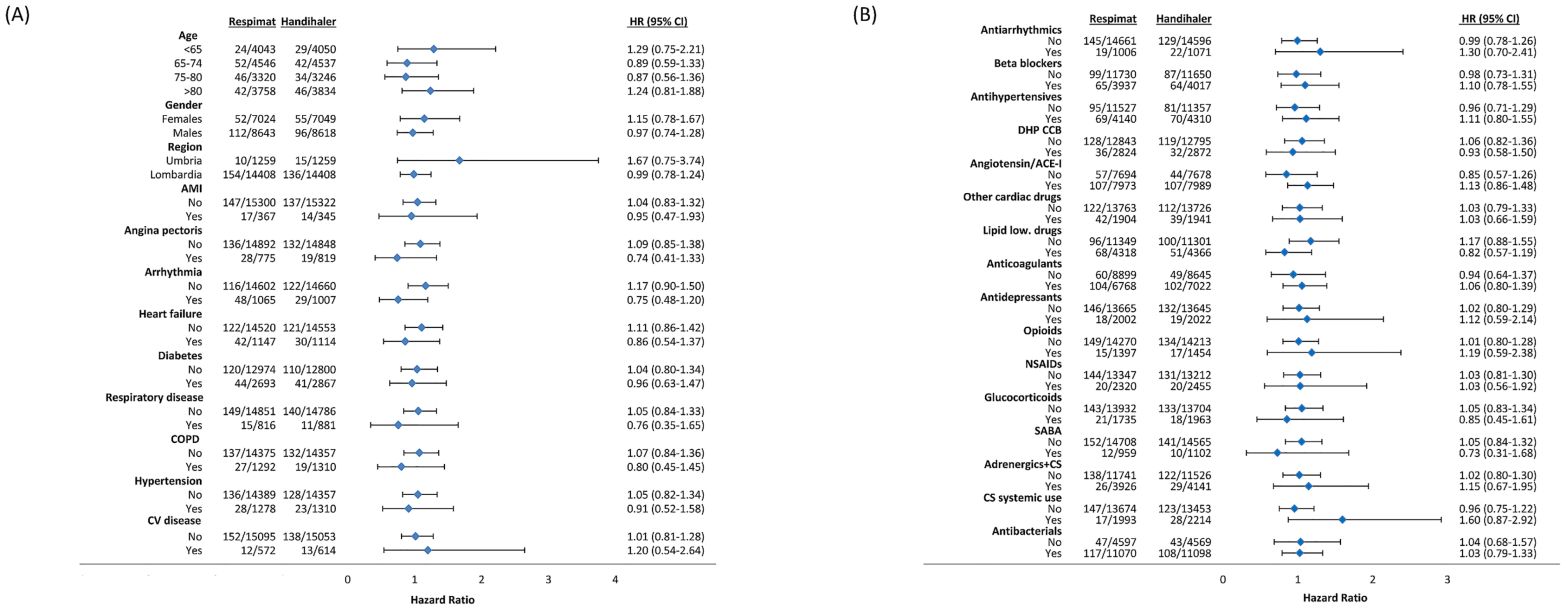
**(a). Hazard ratio of primary outcome by risk factors (patients characteristics and comorbidities) of patients treated with Respimat versus HandiHaler (only risk factors with at least 10 events per group were included in the graphs).** HR: Hazard ratio; CI: Confidence Interval; AMI: Acute myocardial infarction; CV: cerebrovascular. **(b). Hazard ratio of primary outcome by risk factors (drugs) of patients treated with Respimat versus HandiHaler (only risk factors with at least 10 events per group were included in the graphs).** HR: Hazard ratio; CI: Confidence Interval; DHP CCB: dihydropyridine calcium channel blockers; ACE: angiotensin-converting-enzyme; low.: lowering; NSAIDs: nonsteroidal anti-inflammatory drugs; SABA: short-acting beta-agonist; CS: corticosteroid.

No underlying risk factor present in Respimat or HandiHaler users was found to be associated with the primary outcome (Fig 2a and 2b).

Similarly, no differences on primary outcome emerged between Respimat and HandiHaler considering the patients populations excluded from TIOSPIR trial (S3 Table).

### Sensitivity analyses

No risk differences were highlighted when analyzing the primary outcome by calendar period and by fixed follow-up time (S4 and S5 Tables). No difference in terms of in-hospital mortality was observed between Respimat and HandiHaler (S6 Table).

## Discussion

### Principal statement of findings

In this observational cohort study conducted on a large sample of tiotropium users from the routine clinical practice, we found no difference between the incident users of the two formulations of tiotropium (Respimat or HandiHaler) in terms of important acute cardiovascular events, cerebrovascular events and heart rhythm disorders. This result was confirmed in several subgroup analyses specifically designed to evaluate the occurrence of events in patients presenting risk factors that represented exclusion criteria in the TIOSPIR trial.

### Comparison with other studies

Our study confirmed the findings of the TIOSPIR trial and provided reassuring safety data with regard to the use of the Respimat formulation in the general population. In addition, we demonstrated that tiotropium Respimat was as safe as tiotropium HandiHaler also in a population of naïve users. One of the criticisms to the TIOSPIR trial was in fact that it included a large patient population already on treatment with tiotropium (HandiHaler), and thus possibly more tolerant to the anticholinergic effects. A post hoc analysis conducted on a subgroup of TIOSPIR patients who were naïve to anticholinergics showed a similar safety and efficacy profile for both formulations [22]; however, the definition of naïveté was relatively weak (i.e. patients had to be anticholinergic free during the 2 months before the start of the trial). We adopted a more conservative definition, including only patients without prescriptions of tiotropium in the previous 6 months.

Contrary to the TIOSPIR study, the population enrolled in our cohort also included patients with recent cardiovascular and cerebrovascular events, patients with unstable or severe rhythm disorders as well as patients with glaucoma and renal conditions. Given the characteristics of our population, which was similar to the ones described by Schmiedl et al. and/or Scichilone et al [18,21], we believe that our findings are transferable to other Countries.

With regard to the cardiovascular safety, a pooled safety analysis of trials, including TIOSPIR, did not highlight an increased risk for MACE with Respimat [23]. Nevertheless, an ancillary re-assessment of TIOSPIR data conducted by Loke and colleagues raised specific concerns [17]. The incidence of AMI (fatal and non-fatal) was almost 30% higher in Respimat users, although not statistically significant when analyzed separately by dosage (Respimat 2.5 μg or 5.0 μg). Pooling both Respimat dosages, the risk of AMI (fatal and non-fatal) was on the limit of statistical significance (RR: 1.37, 1.00–1.88). Considering only adjudicated fatal AMI, the risk with Respimat was highest (RR: 3.48, 1.04–11.68) although based of very small number of events (21/11,441 vs 3/5,694 in the Respimat vs HandiHaler group). Our study evaluated several cardiovascular safety outcomes and provided reassuring data since we did not identify an excess of AMI, heart rhythm disorders, or cerebrovascular events with Respimat when the events were analyzed as either singular or composite outcomes.

Another large observational cohort study conducted in primary care in the Netherland compared the mortality of the two formulations and found a 30% increased risk of death in Respimat users [8]. However, a sensitivity analyses adjusted by propensity score on incident users, as well as the exclusion of switchers, did not confirm the increased risk of death with Respimat and the HR estimates moved toward the null. The presence of residual confounding may explain this finding. Although the risk of death was not a primary outcome of our study, we calculated the risk of in-hospital mortality with Respimat in a sensitivity analysis and did not find any difference (adjusted HR: 0.88, 0.74–1.04).

### Strengths and weaknesses of the study

Many potential confounders were identified through multiple database linkage, allowing a high completeness of data, especially with regard to underlying comorbidities and co-medications. Several pre-specified sensitivity analyses were also performed and a great consistency of results was observed. Finally, we also investigated the time relationships between the exposure to Respimat or HandiHaler and the primary outcome and found no difference in the occurrence of primary outcome events over time.

We were not able to control our estimates for some potential confounding factors such as smoking status, BMI, socioeconomic status, as well as dosage/posology, which may be associated with the outcomes under study. Moreover, direct clinical information on the time to first COPD diagnosis and the severity of COPD, such as the GOLD stage and FEV1 status, was not available and we used an indirect measure of the COPD severity based on use of respiratory medications, hospitalization for COPD exacerbation or pneumonia. An additional limitation of our study is the relatively short median time of tiotropium use in comparison with the TIOSPIR trial; however, according to a plausible mechanism of action the observation time was consistent with the occurrence of acute events [15].

As with other observational studies based on routinely collected data, an exposure misclassification may have occurred if not all the tiotropium packages prescribed to (and received by) patients were in fact administered. However, such misclassification is expected to be non-differential between the groups. As for other studies that used prescription databases, a limitation of our analysis is that the identification of incident users was based on pharmacy records. The timing of dispensing would affect the inclusion in the incident cohort. We had no information on the actual diagnoses for which the concomitant drugs were used, and the prescription has been interpreted as a proxy indicator for a variety of underlying conditions.

## Conclusions

In conclusion, the results from this large cohort study conducted on incident tiotropium users showed that the two formulations tiotropium (Respimat and HandiHaler) share a similar safety profile, thus confirming in a more heterogeneous population what previously observed in in the TIOSPIR trial.

## Supporting information

S1 Fig Kaplan-Meier curves for the primary outcomes by tiotropium formulations. (DOCX)

S1 Table Outcome definition. (DOCX)

S2 Table Definition of confounders. Part a. Hospitalization (at least one episode in the previous 12 months) and/or drugs (at least two prescriptions in the previous 12 months). Part b. Drugs (at least two prescriptions in the previous 12 months)
(DOCX)

S3 Table Hazard ratio of primary outcome (AMI or heart rhythm disorders) for specific subgroups of patients at higher risk treated with Respimat versus HandiHaler. (DOCX)

S4 Table Hazard ratio of primary outcome (AMI or heart rhythm disorders) in patients treated with Respimat vs HandiHaler by calendar period. (DOCX)

S5 Table Hazard ratio of primary outcome (AMI or heart rhythm disorders) in patients treated with Respimat vs HandiHaler by fixed follow-up period. (DOCX)

S6 Table Hazard ratio of in-hospital mortality in patients treated with Respimat vs HandiHaler. (DOCX)
