# Supplementary material for: Cardiovascular safety of tiotropium Respimat vs HandiHaler in the routine clinical practice: A population-based cohort study
Source: PLoS One. 2017 Apr 21;12(4):e0176276. doi: 10.1371/journal.pone.0176276 (PMC5400270; doi:10.1371/journal.pone.0176276)
Supplement: S1 Fig — (DOCX) [file pone.0176276.s001.docx]

**S1 Fig.** Incidence rate of primary outcomes by tiotropium formulations
