# Supplementary material for: Cardiovascular safety of tiotropium Respimat vs HandiHaler in the routine clinical practice: A population-based cohort study
Source: PLoS One. 2017 Apr 21;12(4):e0176276. doi: 10.1371/journal.pone.0176276 (PMC5400270; doi:10.1371/journal.pone.0176276)
Supplement: S1 Table — (DOCX) [file pone.0176276.s002.docx]

**S1 Table.** Outcome definition

| **Events** | | **ICD-9 Code** | **Notes** |
| --- | --- | --- | --- |
| **Cardiovascular disease** | Acute myocardial infarction | 410 |  |
|  | Sudden death, cause unknown | 798.1; 798.2; 798.9 |  |
|  | Heart failure | 428 |  |
|  | Angina pectoris | 413 |  |
|  | Ill-defined heart disease | 429.1; 429.2; 429.7; 429.8; 429.9 |  |
|  | Other chronic ischemic heart disease | 414 |  |
|  | Other acute ischemic heart disease | 411 |  |
|  | Cardiomyopathy | 425 |  |
|  | Operations on vessels of heart | Procedure code: 36 | Main and secondary procedures |
| **Cerebrovascular disease** |  | 430-438 |  |
| **Rhythm disorders** | Conduction disorders | 426 |  |
|  | Cardiac dysrhythmias | 427 |  |
|  | Heart rhythm procedures | Procedure code: 37 | Main and secondary procedures |

ICD: international classification of diseases
