# Supplementary material for: Cardiovascular safety of tiotropium Respimat vs HandiHaler in the routine clinical practice: A population-based cohort study
Source: PLoS One. 2017 Apr 21;12(4):e0176276. doi: 10.1371/journal.pone.0176276 (PMC5400270; doi:10.1371/journal.pone.0176276)
Supplement: S2 Table — Part a. Hospitalization (at least one episode in the previous 12 months) and/or drugs (at least two prescriptions in the previous 12 months). Part b. Drugs (at least two prescriptions in the previous 12 months) (DOCX) [file pone.0176276.s003.docx]

**S2 Table.** Definition of confounders

**Part a.** Hospitalization (at least one episode in the previous 12 months) and/or drugs (at least two prescriptions in the previous 12 months)

|  | **ICD-9 code** | **ATC code** |
| --- | --- | --- |
| Acute myocardial infarction | 410; 412 |  |
| Acute ischemic heart disease | 411 |  |
| Angina pectoris | 413; 414 |  |
| Cardiomyopathy | 425 |  |
| Arrhythmia | 426; 427 |  |
| Heart rhythm procedures | Procedure code: 37 |  |
| Heart failure | 428 |  |
| Ill-defined heart disease | 429.1; 429.2; 429.7; 429.8; 429.9 |  |
| Cardiogenic shock | 785.51 |  |
| Diseases of pulmonary circulation | 415-417 |  |
| Diabetes mellitus and/or antidiabetic drugs | 250 | A10 |
| Acute kidney failure | 584 |  |
| Chronic kidney diseases | 585 |  |
| Neoplasms | 140-239 |  |
| Respiratory diseases, excl. COPD | 490-496 (excl. 491.21; 493.22; 493.92) |  |
| Chronic obstructive pulmonary disease (COPD) | 491.21; 493.22; 493.92 |  |
| Disorders of lipoid metabolism | 272.0-272.4 |  |
| Operations on vessels of heart | Procedure code: 36 |  |
| Venous thromboembolism | 444; 451; 452; 453 |  |
| Hypertensive diseases | 401-405 |  |
| Cerebrovascular diseases | 433; 434; 436; 437; 440; 441; 442 |  |
| Transient mental disorders and/or antipsychotic drugs | 293-298; 300; 301 | N05A |
| Dementias and/or anti-dementia drugs | 290; 331.0 | N06D |
| Parkinson's disease and/or antiparkinson drugs | 332 | N04 |
| Epilepsy | 345 |  |
| Glaucoma and/or antiglaucoma drugs | 365 | S01ED; S01E |
| Pneumonia | 480-486 |  |

**Part b.** Drugs (at least two prescriptions in the previous 12 months)

|  | **ATC code** |
| --- | --- |
| Antiarrhythmics class I and III | C01B |
| Beta blockers | C07 |
| Antihypertensives and/or  diuretics | C02-C03 |
| Dihydropyridine CCB | C08CA |
| Non Dihydropyridine CCB | C08D |
| Angiotensin receptor blockers and ACE-I | C09 |
| Other cardiac drugs | C01A; C01C-C01E |
| Lipid lowering drugs | C10 |
| Anticoagulants | B01A |
| Antidepressants | N06A |
| Opioids | N02A |
| NSAIDs | M01A |
| Antibacterials | J01 |
| Glucocorticoids | R03BA |
| Anticholinergics | R03BB |
| LABA (Salmeterol; Formoterol; Indacaterol) | R03AC12; R03AC13; R03AC18 |
| SABA (Salbutamol; Terbutaline; Fenoterol) | R03AC02; R03AC03; R03AC04 |
| Leukotriene receptor antagonists | R03DC |
| Xanthines | R03DA |
| Adrenergics in combination with corticosteroids or other drugs, excl. anticholinergics | R03AK |
| Adrenergics in combination with anticholinergics | R03AL |
| Antiallergic agents, excluding corticosteroids | R03BC |
| Other systemic drugs for obstructive airway diseases | R03DX |
| Corticosteroids for systemic use | H02 |

ICD: international classification of diseases; ATC: anatomic therapeutic chemical; CCB: calcium channel blocker; ACE-I: angiotensin converting enzyme inhibitor; NSAIDs: non-steroidal anti-inflammatory drugs; LABA:  Long-Acting Beta Agonist; SABA: short-acting beta-agonist
