# Supplementary material for: Cardiovascular safety of tiotropium Respimat vs HandiHaler in the routine clinical practice: A population-based cohort study
Source: PLoS One. 2017 Apr 21;12(4):e0176276. doi: 10.1371/journal.pone.0176276 (PMC5400270; doi:10.1371/journal.pone.0176276)
Supplement: S3 Table — (DOCX) [file pone.0176276.s004.docx]

**S3 Table.** Hazard Ratio of primary outcome (AMI or heart rhythm disorders) for specific subgroups of patients at higher risk treated with Respimat versus HandiHaler

|  | **Unmatched cohort analysis** | | | **Propensity score matched analysis** | | |
| --- | --- | --- | --- | --- | --- | --- |
|  | **No of events/Total No of patients** | | Unadjusted HR  (95% CI) | **No of events/Total No of patients** | | Adjusted HR  (95% CI) |
|  | Respimat | HandiHaler |  | Respimat | HandiHaler |  |
| Rhythm disorders | 87/5,791 | 391/19,836 | 0.84 (0.67-1.06) | 86/5,370 | 93/5,463 | 0.99 (0.74-1.33) |
| Hearth disease | 106/7,174 | 471/25,742 | 0.89 (0.72-1.10) | 111/6,868 | 106/7,074 | 1.03 (0.79-1.35) |
| Acute and/or chronic kidney failure | 7/501 | 60/2,306 | 0.66 (0.30-1.45) | 7/492 | 17/512 | 0.52 (0.22-1.26) |
| Frail population | 63/3,895 | 265/15,148 | 1.04 (0.79-1.38) | 61/3,768 | 63/3,857 | 1.17 (0.82-1.66) |

**Rhythm disorders:** Arrhythmia (ICD-9 code 426; 427) and/or Heart rhythm procedures (ICD-9 Procedure code 37) and/or Glaucoma and/or antiglaucoma drugs (ICD-9 code 365; ATC S01ED; S01E) and/or Antiarrhythmics class I and III (ATC C01B) and/or Beta blockers (ATC C07)

**Hearth disease:** Acute myocardial infarction (ICD-9 code 410; 412) and/or Acute ischemic heart disease (ICD-9 code 411) and/or Anticoagulants (ATC B01A)

**Acute and/or chronic kidney failure:** Acute kidney failure (ICD-9 code 584) and/or Chronic kidney diseases (ICD-9 code 585)

**Frail population:** Age>75 years and [Acute myocardial infarction (ICD-9 code 410; 412) or Anticoagulants (ATC B01A)]
