# Supplementary material for: Cardiovascular safety of tiotropium Respimat vs HandiHaler in the routine clinical practice: A population-based cohort study
Source: PLoS One. 2017 Apr 21;12(4):e0176276. doi: 10.1371/journal.pone.0176276 (PMC5400270; doi:10.1371/journal.pone.0176276)
Supplement: S4 Table — (DOCX) [file pone.0176276.s005.docx]

**S4 Table.** Hazard Ratio of primary outcome (AMI or heart rhythm disorders) in patients treated Respimat vs HandiHaler by year

| **Year** | **Propensity score matched analysis** | | |
| --- | --- | --- | --- |
|  | **No of events/Total No of patients** | | **Adjusted HR** |
|  | **Respimat** | **HandiHaler** | **(95% CI)** |
| **2011** | 22/3,072 | 43/4,684 | 0.95 (0.53-1.73) |
| **2012** | 77/7,540 | 100/8,374 | 0.90 (0.64-1.27) |
| **2013** | 52/5,003 | 21/2,636 | 1.49 (0.85-2.64) |
