# Supplementary material for: Cardiovascular safety of tiotropium Respimat vs HandiHaler in the routine clinical practice: A population-based cohort study
Source: PLoS One. 2017 Apr 21;12(4):e0176276. doi: 10.1371/journal.pone.0176276 (PMC5400270; doi:10.1371/journal.pone.0176276)
Supplement: S5 Table — (DOCX) [file pone.0176276.s006.docx]

**S5 Table.** Hazard Ratio of primary outcome (AMI or heart rhythm disorders) in patients treated Respimat vs HandiHaler by fixed exposure period

| **Exposed period**  **(days)** | **Propensity score matched analysis** | | |
| --- | --- | --- | --- |
|  | **No of events/Total No of patients** | | **Adjusted HR** |
|  | **Respimat** | **HandiHaler** | (95% CI) |
| **30** | 70/15,667 | 62/15,667 | 1.14 (0.81-1.60) |
| **45** | 98/15,667 | 87/15,667 | 1.14 (0.86-1.52) |
| **75** | 120/15,667 | 120/15,667 | 1.06 (0.82-1.36) |
