# Supplementary material for: Cardiovascular safety of tiotropium Respimat vs HandiHaler in the routine clinical practice: A population-based cohort study
Source: PLoS One. 2017 Apr 21;12(4):e0176276. doi: 10.1371/journal.pone.0176276 (PMC5400270; doi:10.1371/journal.pone.0176276)
Supplement: S6 Table — (DOCX) [file pone.0176276.s007.docx]

**S6 Table.** Hazard Ratio of in-hospital mortality in patients treated Respimat vs HandiHaler

|  | **Unmatched cohort analysis** | | | **Propensity score matched analysis** | | |
| --- | --- | --- | --- | --- | --- | --- |
|  | **No (%) of events** | | Unadjusted HR  (95% CI) | **No (%) of events** | | Adjusted HR  (95% CI) |
|  | Respimat  (n=15,937) | HandiHaler  (n=53,088) |  | Respimat  (n=15,667) | HandiHaler  (n=15,667) |  |
| **In-hospital mortality** | 244 (1.53) | 1,213 (2.28) | 0.72 (0.63-0.83) | 242 (1.54) | 305 (1.95) | 0.88 (0.74-1.04) |
